# Peek

**Published:** 2015

Peek, the Portable Eye Examination Kit, is a set of diagnostic tools that allows eye care workers to use a smartphone to screen eye patients. It makes use of ‘cloud’-based systems to enable data sharing, referral and follow-up of patients.

The Peek team has developed tests for visual acuity, contrast, colour, visual fields, and childhood vision.

Peek Acuity, the visual acuity test (soon to be released on Android phones in late 2015/early 2016), is shown in a validation study published in *JAMA Ophthalmology* to be reliable, accurate and fast. It has since been used by teachers in a school screening programme in Kenya in which over 20,000 children were screened in two weeks.

Peek is also working closely with partners to provide population-based survey tools, outreach systems and educational materials – all of which will be done using a smartphone.

Peek apps will be free to download from the Google Play store once ready for release. To keep updated on our research, release dates and news, please sign up to our newsletter at **www.peekvision.org**

**Figure:**
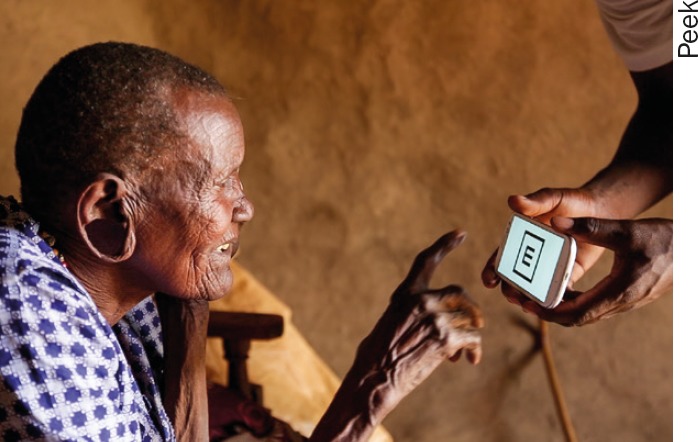
A community health worker uses Peek to measure someone's vision in their own home. KENYA

